# Enzyme-linked immunosorbent assay (ELISA) immunoassaying versus microscopy: advantages and drawbacks for diagnosing giardiasis

**DOI:** 10.1590/S1516-31802005000600006

**Published:** 2005-11-01

**Authors:** Alaíde Mader Braga Vidal, Wilson Roberto Catapani

**Keywords:** Giardiasis, Giardia lamblia, Parasitic intestinal diseases, Clinical laboratory techniques, ELISA, Giardíase, *Giardia lamblia*, Enteropatias parasitárias, Técnicas de laboratório clínico, ELISA

## Abstract

**CONTEXT AND OBJECTIVE:**

Giardiasis is common in Brazil. For laboratory diagnosis, the method most utilized is microscopic examination of fecal samples, but the immunoenzymatic method is also available. The aim of this work was to verify the advantages and drawbacks of immunoassaying versus microscopy for diagnosing *Giardia lamblia*, when a single fecal sample is analyzed.

**DESIGN AND SETTING:**

Prospective, double-blind study at the parasitology laboratory of Faculdade de Medicina da Fundação ABC.

**METHODS:**

Samples were prepared according to the traditional sedimentation (Hoffman, Pons and Janer) and Faust methods. Results were deemed positive when *Giardia lamblia* was found by one or both methods. The Prospect ELISA kit was used for detecting *Giardia lamblia*-specific antigen, in accordance with the manufacturer's instructions. Results were expressed on a visual scale as negative or positive (+, ++, +++ or ++++).

**RESULTS:**

The ELISA test was positive even when a significant proportion of corresponding samples examined by microscopy were negative. This trend was statistically significant (p < 0.001). The overall concordance of results between the ELISA test and microscopic examination of single samples was only moderate (0.50 by kappa test).

**CONCLUSION:**

The ELISA test is useful when just searching for *Giardia lamblia*, because of its high sensitivity. For daily practice, we recommend microscopy, which is much cheaper and can also detect other parasites. The low positivity of single samples in this method can be overcome by using three samples, as recommended by most authors.

## RESUMO

**CONTEXTO E OBJETIVO:**

A giardíase é comum no Brasil. Para o diagnóstico laboratorial, o método mais empregado é o exame microscópico de amostras fecais. O método imunoenzimático (ELISA) também é utilizado. O objetivo deste trabalho é verificar as vantagens e desvantagens do método microscópico quando comparado ao imunoenzimático para o diagnóstico de *Giardia lamblia* em uma única amostra fecal.

**TIPO DE ESTUDO E LOCAL:**

Estudo prospectivo, duplo cego, no Laboratório de Parasitologia, Faculdade de Medicina da Fundação ABC.

**MÉTODOS:**

As amostras foram preparadas para exame de acordo com os tradicionais métodos de sedimentação (Hoffman, Pons e Janer) e Faust. Um resultado positivo significa o encontro de *Giardia lamblia* por um dos métodos ou ambos. O kit Prospect ELISA foi utilizado para detecção do antígeno específico de *Giardia lamblia*, de acordo com as instruções do fabricante. Os resultados foram expressos em escala visual como negativos ou positivos (+, ++, +++ ou ++++).

**RESULTADOS:**

O teste ELISA é positivo mesmo quando uma significante proporção das correspondentes amostras examinadas por microscopia ainda é negativa, sendo esta tendência estatisticamente significante (p < 0,001). A concordância de resultados entre os dois métodos é apenas moderada (0,5 pelo teste kappa).

**CONCLUSÃO:**

O teste ELISA é recomendável quando se busca uma elevada sensibilidade para a detecção de antígenos específicos de *Giardia lamblia*, como em estudos de prevalência. Para a prática diária, recomendamos o método microscópico, que tem custo muito menor e pode detectar outros parasitas na mesma amostra. A baixa taxa de positividade do método no exame de uma única amostra pode ser contornada pelo exame de três amostras, como recomendado pela maioria dos autores.

## INTRODUCTION

Giardiasis is common worldwide, and 10% to 15% of the population is infected, even in first-world countries.^1^ In Brazil, unfortunately, there is still too much neglect of this subject, considering that data from local researchers show high prevalence rates of *Giardia lamblia* infection in several places in the country. Studies carried out in São Paulo,^2–9^ Minas Gerais,^10^ Paraíba,^11^ Rio Grande do Norte,^12^ the Federal District^13^ and Sergipe^14^ have demonstrated prevalence rates in the range of 9% to 50%, especially among children of up to four years old.

For laboratory diagnosis, the method most utilized is microscopic examination of fecal samples. Nevertheless, the immunoenzymatic method is also available. This assay is capable of detecting small quantities of fecal parasitic antigens, even in mild infections. The 65-kDa *Giardia*-specific antigen 65 (GSA 65) glycoprotein was considered to be the antigen of interest. It is present in the cysts and trophozoites of *Giardia lamblia* and is very specific to this parasite. It is the main antigen found in the feces of individuals infected with *Giardia lamblia*, and it has been used for immunodiagnosis.^15^

The earliest immunoenzymatic assays performed showed sensitivity of 92% and specificity of 98%, with the inconveniencing factor that they needed to be performed using fresh stools.^16^ In 1989, it became possible to apply specific antigens against the *Giardia lamblia* cyst wall, such as GSA 65, and this increased the sensitivity from 96% to 99% and the specificity from 96% to 100%.^17^ The commercial kits available today allow the utilization of preserved samples, thus making the process easier. However, the limiting factor in using this technology is its high cost in comparison with traditional microscopy methods.

## OBJECTIVE

This study aimed to verify the efficacy, advantages and drawbacks of immunoassaying versus microscopy for diagnosing *Giardia lamblia*, when single fecal samples are analyzed.

## METHODS

### Type of study

Prospective, double-blind study.

### Setting

The study was carried out at the parasitology laboratory of Faculdade de Medicina da Fundação ABC.

### Sample

A total of 142 fecal samples (one sample from each of 142 patients) were analyzed using both immunoassaying and microscopy.

All the samples were prepared for examination according to the traditional sedimentation method (Hoffman, Pons and Janer) and the Faust method. For microscopic examination, the fecal samples were prepared and analyzed under the microscope as soon as they reached the laboratory. Microscope slides were always examined by the same very experienced person. Results were deemed positive when *Giardia lamblia* was found by one or both of the methods.

For the enzyme-linked immunosorbent assay (ELISA) method, aliquots taken from the same fecal samples were stored at −20° C, for periods of no more than 30 days before their utilization. The Prospect ELISA kit was used for detecting *Giardia lamblia*-specific antigen (Alexon-Biobras, Belo Horizonte, Minas Gerais). The entire procedure was carried out in accordance with the manufacturer's instructions. The results were expressed on a visual scale as negative, +, ++, +++ or ++++.

### Statistical methods

The chi-squared test for trend was used to verify the association between the results from the microscopic and ELISA methods. The kappa test was used to assess the concordance of results from the two techniques. For this purpose, all the positive results (+, ++, +++ and ++++) from the ELISA test were simply taken to be “positive” results, regardless of the intensity of the reaction. The kappa test was performed using the True Epistat (4^th^ edition, Richardson, Texas, USA) statistical software, which assumes a normal distribution for the values and calculates a Z test.

## RESULTS

Table 1 shows that, out of the 142 samples, 135 were negative by microscopic examination, whereas 130 were negative by the ELISA test. Among the 12 positive samples by ELISA, two displayed weak reactions (+), and these were negative by microscopy. Among the three ++ positive samples by ELISA, one was also positive by microscopy and the other two were negative. Five samples were classified as +++ positive by ELISA and, among these, three were positive and two negative by microscopy. Finally, two samples gave strongly positive ELISA tests (++++) and, among these, one was positive and the other negative by microscopy.

**Table 1.** Number of positive samples for *Giardia lamblia* in each method of giardiasis diagnosis and the intensity of the color reaction in the enzyme-linked immunosorbent assay (ELISA) method in 142 fecal samples

ELISA Number of samples/reaction intensity	Positive microscopy: number of samples	Negative microscopy: number of samples
130 negatives	2	128
2 positives (+)	0	2
3 positives (++)	1	2
5 positives (+++)	3	2
2 positives (++++)	1	1
**Total**	**7**	**135**

*Chi-squared test for trend: p < 0.0001.*

Among the 130 negative samples by ELISA, 128 were also negative by microscopy. However, two were considered positive by this method.

Table 1 shows that when the ELISA test is positive, a proportion of the corresponding samples is still negative by microscopy. This pattern is observed even when the ELISA test is strongly positive, and this trend is highly significant by the chi-squared test. Table 2 shows the concordance index between the ELISA and the microscopic method, obtained by the kappa test.

**Table 2.** Concordance between the results from the enzyme-linked immunosorbent (ELISA) test and the microscopic examination of single samples, as assessed by the kappa test in the examination of 142 fecal samples for giardiasis

Kappa	Standard error	z score	^p^
0.5	0.074	6.70	< 0.001

## DISCUSSION

The diagnosing of giardiasis is very frequently dependent on the sequential examination of several fecal samples by a skilled person. In many cases, the parasite is not revealed by the examination of a single fecal sample. Hiatt et al.^18^ recommended that three samples per patient should be examined; otherwise the diagnosis rate might be significantly underestimated. According to these authors, the diagnostic yield increased by 11.3% for *Giardia lamblia* and 22.7% for *Entamoeba histolytica*, when three samples were examined instead of only one. The ELISA technique is fast and easy to perform for many samples at a time.

Other researchers, in developing an ELISA method for *Giardia lamblia*, showed that there was no difference in the antigenic activity of the system between the supernatant obtained from centrifuged samples and small samples of feces refrigerated at 4°C for up to two weeks.^16^ Also, repeated thawing and freezing of the samples (up to fifteen times) did not interfere with their antigenicity. In our study, once the samples were frozen, they were only thawed out to perform the ELISA test.

Green et al.^17^ developed another ELISA method for the detection of fecal antigens, in which the results could be evaluated in a visual colorimetric manner, thereby obtaining sensitivity greater than 98% and specificity of 100%. Since then, several kits have been developed. Garcia and Shimizu^19^ tested nine of these kits and found sensitivities ranging between 94% and 99%, with specificity of 100% for all of them, including the kit used in our experiment (Prospect ELISA for *Giardia lamblia*, Alexon Inc.). Mank et al.^20^ compared two commercial kits for detecting *Giardia lamblia* fecal antigens and reported that testing single fecal samples by this technique resulted in a diagnostic ratio similar to what was obtained when two separate samples were examined by microscopy techniques.

Schunk et al.^21^ tested an ELISA kit for detecting *Giardia lamblia* and reported no false positive results caused by the presence of other protozoa or helminthes in the feces. Among the 276 patients included in their study, 17.4% had at least one other protozoon or helminthe: twenty-four had *Blastocystis hominis*, twelve had *Entamoeba coli* and ten had *Endolimax nana*, while others had *Isospora belli*, *Necator americanus*, *Strongyloides stercoralis* and *Ascaris lumbricoides*. No patient among these reacted positively in the ELISA test. On the other hand, this indicates an advantage that microscopic examination has over ELISA: although the latter is very specific, it will not detect other parasites that the patient might have and which would be identified by microscopy. Among our patients, there were individuals who had other parasites that were identified by microscopic examination. Some patients who were negative for *Giardia lamblia* had *Ascaris lumbricoides*, *Hymenolepis nana, Strongyloides stercoralis, Schistosoma mansoni* and *Ancilostoma* that were detected under the microscope. Among the *Giardia lamblia-*positive patients, some had *Trichuris trichiura.*

Our results (Table 1) have provided a comparison between the detection of *Giardia lamblia* by the microscopy technique versus the ELISA method, in single fecal samples. The results obtained may suggest that the ELISA test has greater sensitivity. This test might be able to detect minimal amounts of antigen and hence give a positive result even when the parasite load is slight. In such cases, several samples examined by microscopy would probably be required until one resulted positive. Therefore, the ELISA test is already positive even when a significant proportion of the corresponding samples examined by microscopy are negative, and this association is statistically significant (p < 0.001). In Table 2, the discrepancy between the results from the ELISA test and the microscopy technique can be seen, and this is reflected in the overall moderate agreement between the two methods, when following the recommendations for kappa test interpretation from Landis and Koch (1977).^22^

The occurrence of two patients who were positive by microscopy, among the patients who were negative by the ELISA method, may correspond to false positives due to examiner error. Even when the examiner is experienced, there is the possibility of a mistake in the identification, especially of the cystic forms.

With regard to the cost, each test performed by the ELISA method in Brazil had an estimated cost of 6.70 United States dollars (circa 18.76 Brazilian reais), whereas the cost of a test utilizing the Hoffman technique is about 0.05 Brazilian reais (US$ 0.02) and about 0.30 Brazilian reais (US$0.10) by the Faust technique.

## CONCLUSION

We agree with Machado's opinion that the ELISA test should be used for epidemiological studies that have the strict goal of detecting giardiasis,^23^ because of its very great sensitivity. However, for daily practice, the low cost and capacity for detecting other parasites are factors that strongly recommend the utilization of three fecal samples examined by microscopic methods. This is especially so in our country, where it is not uncommon to find more than one parasite in the same patient (Table 3).

**Table 3.** Comparison of advantages and disadvantages of ELISA and microscopy methods for the diagnosis of giardiasis

ELISA Prospect *Giardia*	Microscopic methods
Highly sensitive and specific for *Giardia lamblia* in single fecal samples	Need for several samples to be examined. Patients must go to the laboratory three times.
Samples can be stored for up to 30 days.	Need to be performed on fresh feces.
Does not identify associated parasites in the sample.	Can identify other parasites in the sample.
High cost	Low cost

*ELISA = enzyme-linked immunosorbent assay.*
